# Numerical Comparison of Prediction Models for Aerosol Filtration Efficiency Applied on a Hollow-Fiber Membrane Pore Structure

**DOI:** 10.3390/nano8060447

**Published:** 2018-06-19

**Authors:** Pavel Bulejko

**Affiliations:** 1 Heat Transfer and Fluid Flow Laboratory, Faculty of Mechanical Engineering, Brno University of Technology, Technická 2, 616 69 Brno, Czech Republic; pavel.bulejko@vut.cz; Tel.: +420-541-144-912; 2 MemBrain s.r.o. Pod Vinicí 87, 471 27 Stráž pod Ralskem, Czech Republic

**Keywords:** hollow-fiber membrane, aerosol, filtration efficiency, interception, inertial impaction, diffusion

## Abstract

Hollow-fiber membranes (HFMs) have been widely applied to many liquid treatment applications such as wastewater treatment, membrane contactors/bioreactors and membrane distillation. Despite the fact that HFMs are widely used for gas separation from gas mixtures, their use for mechanical filtration of aerosols is very scarce. In this work, we compared mathematical models developed for the prediction of air filtration efficiency by applying them on the structural parameters of polypropylene HFMs. These membranes are characteristic of pore diameters of about 90 nm and have high solidity, thus providing high potential for nanoparticle removal from air. A single fiber/collector and capillary pore approach was chosen to compare between models developed for fibrous filters and capillary-pore membranes (Nuclepore filters) based on three main mechanisms occurring in aerosol filtration (inertial impaction, interception and diffusion). The collection efficiency due to individual mechanisms differs significantly. The differences are caused by the parameters for which the individual models were developed, i.e., given values of governing dimensionless numbers (Reynolds, Stokes and Peclet number) and also given values of filter porosity and filter fiber diameter. Some models can be used to predict the efficiency of HFMs based on assumptions depending on the conditions and exact membrane parameters.

## 1. Introduction

Air filtration is the most frequently used method for airborne particulate matter mitigation [1,2]. Dust, allergens, microorganisms, welding fumes and combustion-generated particles have been of growing interest due to associated health concerns. It has been found that there is a direct relationship between increased concentrations of airborne particles and human health disorders [3,4,5,6,7]. With regard to increasing nanotechnology applications, airborne nanoparticles have been of growing interest [8,9,10,11] as well as technologies for their mitigation. This mainly entails the development of various filtration materials based on nanofibers or a membrane structure [12,13,14,15]. The former has recently been a subject of many works while the latter was of interest mainly when dealing with capillary pore membranes (CPMs, so called Nuclepore filters) used for measuring workplace exposure [16,17,18]. Predicting the performance of such filters/membranes was of great concern in terms of their particle removal efficiency, including minimum efficiency, most penetrating particle size (MPPS) and pressure drop. Therefore, many works have been carried out to develop mathematical expressions to calculate filtration efficiency in relation to particle size. Different models were developed for efficiency predictions of fibrous filters and CPMs. While filtration mechanisms of a CPM relates mainly to surface filtration and sieving, fibrous filters separate particles mainly via inertial impaction, interception, Brownian motion (diffusion), gravitational settling and electrostatic deposition in electret filters [19].

Hollow-fiber membrane (HFM) is a special type of membrane geometry characteristic of compactness: It contains a large surface area in a small volume. Thus far, all applications of porous HFMs could have been found mostly in water treatment applications [20,21,22,23] and non-porous HFMs in gas separation and heat exchangers [24,25,26]. Such geometry ensures a high surface area necessary for mass/heat transfer applications. HFMs can have a symmetric or asymmetric porous structure depending on the way of preparation. A symmetric structure of HFM can be achieved in a number of ways, such as via dry stretching of extruded polymeric hollow fiber [27]. An asymmetric structure is obtained when a thin skin layer is coated in a HFM surface (shell side or lumen side) or can be integral i.e., the pore size decreases in the direction of membrane surface [28,29,30].

The number of publications concerning the use of HFMs in gas filtration applications is very scarce. There have recently been only three publications focused on the separation of solid particles from air using HFMs. Wang et al. [31] prepared asymmetric polyvinylidene fluoride-polyethylene glycol (PVDF-PEG) HFMs and tested for air filtration performance against ultrafine polydisperse NaCl aerosol with a geometric average particle size of 30 nm. The results showed a high filtration efficiency of 99.999%. In the other work, Li et al. [32] focused on design and characterization of HFMs based on poly(ether sulfone) prepared via dry-jet wet spinning. They prepared an asymmetric HFM composed of a fibrous-like porous substrate with a membrane sieve-like layer on its surface and achieved an efficiency of 99.995% when challenged with less than 300 nm ammonium sulfate particulates. Lastly, the authors of the third publication [27] used symmetric polypropylene HFMs to separate polydisperse nanoaerosol generated using incense stick burning. They discovered that the HFMs have MPPS in range of 34–40 nm with a MPPS efficiency of 79–87% depending on flowrate. Furthermore, the results showed efficiency levels higher than 99% for particles above 60 nm and remain unchanged with the flowrate. Potential applications for HFM as air filters are mainly in low volume applications. Such applications include printing board filters, microelectronics, sterilized water tank ventilation and clean air for sensitive analytical or medical devices. The HFM can provide high efficiency due to its narrow pore size distribution and small pore sizes in the range of 90 nm. Moreover, HFMs can provide long service life due to possibility of simple regeneration.

**Figure 1:**
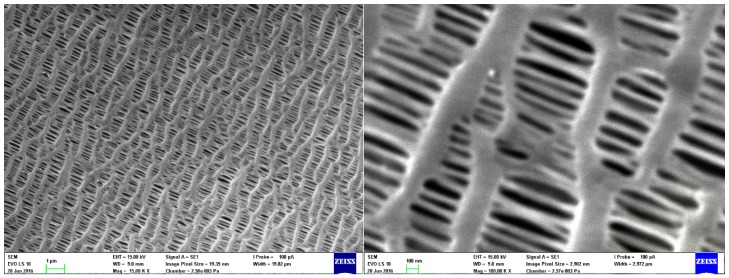
Polypropylene HFM pore structure.

In this work, we applied mathematical models developed for prediction of air filtration efficiency of fibrous filters and CPMs on a HFM pore structure. For calculation, we used parameters of symmetric polypropylene HFM produced by ZENA Membranes s.r.o. [33]. From the porous structure of these HFMs (Figure 1), and comparing with a typical structure of a fibrous filter (see e.g., [12,34,35,36,37,38] and a CPM (see e.g., [39,40,41,42,43]), we can see several similarities but also several main differences. First, the HFM pore structure is composed of longitudinal segments (referred to as collectors) with an average diameter of about 90 nm. These can be considered fibers analogically to fibrous filters. Second, the pore structure contains elliptical pores that are analogical to CPM, which has circular pores. Conversely, HFMs have very high solidity (here 0.48) compared to commercial fibrous filters, which typically have solidity between 0.01 and 0.3 [44]. So with some assumptions, the models for fibrous filters and CPMs can be applied on the HFMs considered in this study. Therefore, the main effort of this work is to compare these models by numerically applying them on HFM assuming that the collection mechanisms are analogical to those considered in fibrous filters and CPMs. Based on single fiber theory developed for fibrous filters, we determined single collector efficiencies (SCE) based on different mechanisms taking place in aerosol filtration. The efficiency results were compared between SCE models for individual mechanisms developed by various researchers. Therefore, this work can also serve as an overview of mathematical models for SCE due to different capturing mechanisms.

## 2. Prediction Models for Air Filtration Efficiency

Air filtration materials or whole air filtration units are mostly evaluated in terms of filtration efficiency and pressure drop. The former describes the ability of a filter unit to remove particles from air stream while the latter one is related mainly to energy requirements. The filtration efficiency *η* is defined as follows:

(1) $$\eta =1-\frac{C_{down}}{C_{up}}$$

where *C_down_* and *C_up_* are the number of particles downstream and upstream of the filter, respectively.

### 2.1. Efficiency Prediction of Fibrous Filters

**Figure 2:**
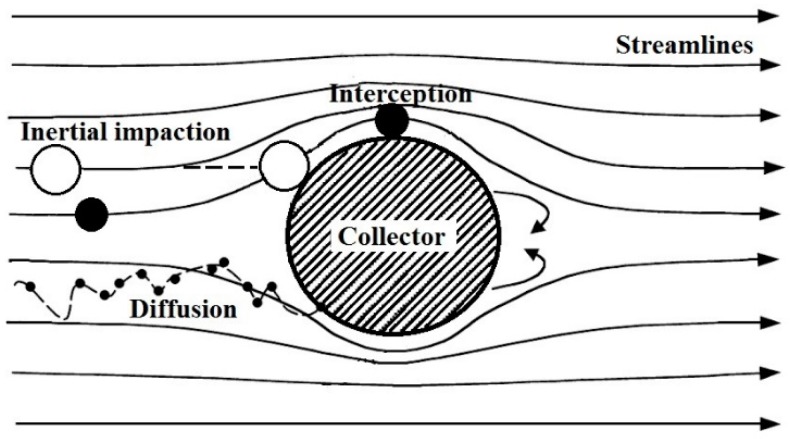
Schematic presentation of individual collection mechanisms at a single collector.

Non-woven fibrous filters are composed of fibers, which are randomly oriented even though the orientation is mostly normal to the airflow. The diameter of fibers is mostly not uniform and can be produced from various mostly polymeric materials. The filtration efficiency of fibrous filters may be predicted based on several parameters and assumption of an idealized filter structure. The formula is as follows [45]:

(2) $$\eta =1-\exp\left[-\frac{4\alpha \eta_{f}Z}{\pi (1-\alpha )d_{f}}\right]$$

where *α*, *η*_f_, *Z* and *d*_f_ are the filter solidity, SCE, filter thickness and average collector diameter, respectively. The total SCE is a sum of contributions from different collection mechanisms and can be written as follows:

(3) $$\eta_{f}=(\eta_{I}+\eta_{R}+\eta_{D})\eta_{A}$$

where *η*_I_, *η*_R_, *η*_D_ and *η*_A_ are the single collector efficiencies due to inertial impaction, interception, diffusion and adhesion, respectively. The filtration theory, which is based on three main mechanisms, inertial impaction, interception and diffusion (Figure 2), does not take into account particle-fiber interaction, i.e., the particle rebound and re-entrainment. Therefore, we used Equation (3) to calculate the SCE based on collision efficiency (sum of collection efficiencies due to impaction interception and diffusion) multiplied by the collection efficiency caused by adhesion effects [46,47].

#### 2.1.1. SCE Due to Brownian Motion

Filtration efficiency due to diffusion (Brownian motion) is a significant part of the overall filtration efficiency. The randomly changing trajectory of very small particles (Figure 2) increases the probability of hitting the collector and their capture by filter. The governing parameter for diffusion mechanism is Peclet number, which is the ratio of convection and diffusion transport rate as follows:

(4) $$Pe=\frac{Ud_{f}}{D}$$

where *U* is the face velocity and *D* is the diffusion coefficient of particle calculated as follows:

(5) $$D=\frac{k_{B}TC_{s}}{3\pi \mu d_{p}}$$

where *k*_B_, *T*, *µ* and *d*_p_ are the Boltzmann constant, absolute temperature, air dynamic viscosity and particle diameter, respectively and *C*_s_ is the Cunningham slip correction factor:

(6) $$C_{s}=1+Kn\left[1.207+0.44\exp\left(-\frac{0.78}{Kn}\right)\right]$$

where *Kn* is the Knudsen number of particle with *λ* as mean free path of gas molecules:

(7) $$Kn=\frac{2\lambda }{d_{p}}$$

Several relationships have been proposed to predict SCE due to diffusion (*η*_D_). For nanoparticles that have high diffusion coefficient, hence smaller Peclet number, Wang et al. [48] gave the following relationship:

(8) $$\eta_{D}=0.84Pe^{-0.43}$$

Equation (8) suggests a lower dependence of diffusion efficiency on the Peclet number, though it is in good agreement with experimental data for whole range of Peclet numbers. Another relationship was proposed by Kirsch and Fuchs [49]:

(9) $$\eta_{D}=2.7Pe^{-2/3}$$

Equations (8) and (9) does not include the effect of flow field distortion at the gas-fiber interface and are independent. Therefore, several researchers proposed different expressions based on theoretical derivation or experimental data. Stechkina et al. [50] proposed following relationship:

(10) $$\eta_{D}=2.9Ku^{-1/3}Pe^{-2/3}+0.62Pe^{-1}$$

while analysis of Pich [51] and Lee and Liu [52] lead to Equations (11) and (12), respectively:

(11) $$\eta_{D}=2.27Ku^{-1/3}Pe^{-2/3}(1+0.62KnPe^{1/3}Ku^{-1/3})$$

(12) $$\eta_{D}=1.6{\left(\frac{1-\alpha }{Ku}\right)}^{1/3}Pe^{-2/3}$$

where *Ku* is the Kuwabara hydrodynamic factor. The Kuwabara factor compensates the flow field distortion around a collector occurring due to its proximity to neighboring fibers. The Kuwabara factor is a dimensionless parameter and depends only on filter solidity *α* for *d*_f_ ≥ 2 µm as follows:

(13) $$Ku=-\frac{\ln\alpha }{2}+\alpha -\frac{\alpha^{2}}{4}-\frac{3}{4}$$

As the slip effect becomes significant for filters with fiber diameter smaller than 2 µm (which is true for HFMs considered in this work), Kirsch and Stechkina [53] recommended adding the Knudsen number of fiber (13) to compensate for the slip effect:

(14) $$Kn_{f}=\frac{2\lambda }{d_{f}}$$

Thus, for a fiber diameter smaller than 2 microns, the relationship for the Kuwabara factor is:

(15) $$Ku=\frac{2\lambda }{d_{f}}-\frac{\ln\alpha }{2}+\alpha -\frac{\alpha^{2}}{4}-\frac{3}{4}$$

The same was proposed for the relationships for diffusion efficiency, i.e., modifying using a correction factor accounting for slip flow when the fiber diameter is in the same magnitude as the mean free path of the gas molecules. Using the work of Lee and Liu [52] as a basis (Equation (12)), Liu and Rubow [54] corrected this model to consider the slip effect as follows:

(16) $$\eta_{D}=1.6{\left(\frac{1-\alpha }{Ku}\right)}^{1/3}Pe^{-2/3}C_{1}$$

where *C*_1_ is a constant calculated as follows:

(17) $$C_{1}=1+0.388Kn_{f}{\left[\left(1-\alpha \right)\frac{Pe}{Ku}\right]}^{1/3}$$

However, efficiencies calculated using Equation (16) might exceed unity for very small particles (low Peclet numbers). Therefore, Payet et al. [55] introduced another correction factor, to get the efficiency for very small particles under unity, as follows:

(18) $$\eta_{D}=1.6{\left(\frac{1-\alpha }{Ku}\right)}^{1/3}Pe^{-2/3}C_{1}C_{2}$$

where *C*_2_ is calculated as follows:

(19) $$C_{2}=\frac{1}{1+1.6{\left(\frac{1-\alpha }{Ku}\right)}^{1/3}Pe^{-2/3}C_{1}}$$

Note that the constant 1.6 in Equation (12) and the other derived based on the same constant may be substituted with a different value (mostly higher value of 2.6 or 2.9) to obtain a better agreement with experimental data. The commonly used single collector theory was developed for the Kuwabara cell model [56]. This model, however, does not consider possible heterogeneities of filter structure (local porosity variations) related to non-uniform fiber distribution or their size polydispersity [57].

#### 2.1.2. SCE Due to Interception

Interception occurs when a particle following fluid streamline flowing around the collector is in a distance of one particle radius from the collector surface (Figure 1). The interception mechanism is governed by the interception parameter *R*, which is the ratio of particle to fiber diameter:

(20) $$R=\frac{d_{p}}{d_{f}}$$

Interception efficiency increases by increasing the interception parameter [58]. Following this, the interception efficiency should be independent of the airflow velocity, which is true for most models developed for SCE due to interception. However, considering the filter fibers as isolated cylinders, the interception efficiency obtained from Lamb’s solution of Navier-Stokes equations [59] is dependent on Reynolds number hence airflow velocity. Langmuir [60] derived this relationship for low Reynolds numbers (Re_f_ < 1) as follows:

(21) ηR=2(1+R)ln(1+R)−(1+R)+1/(1+R)2(2−lnRef)

with Re_f_ as fiber Reynolds number characterizing flow field around a fiber calculated as follows:

(22) Ref=dfUρμ

where *ρ* is the fluid density. Majority of mathematical expressions for interception efficiency are based on the Kuwabara cell model [56] and are independent of fluid velocity. Kirsch and Stechkina gave a complete model for SCE due to interception as follows [53]:

(23) $$\eta_{R}=\frac{1+R}{2Ku}\left[2\ln(1+R)-1+\alpha +{\left(\frac{1}{1+R}\right)}^{2}\left(1-\frac{\alpha }{2}\right)-\frac{\alpha }{2}{\left(1+R\right)}^{2}\right]$$

This is the basic formula for the SCE due to interception based on the Kuwabara flow field. However, it is a rather long and complicated expression, which Lee and Liu reduced to following simpler forms [52]:

(24) $$\eta_{R}=\frac{1-\alpha }{Ku}\frac{R^{2}}{1+R}$$

(25) $$\eta_{R}=0.6\frac{1-\alpha }{Ku}\frac{R^{2}}{1+R}$$

Equation (24) is valid for *R* < 0.2 and *α* < 0.5. With the assumption that fibers are not oriented perpendicular to the flow direction and for non-uniform fiber distribution, Lee and Liu [52] modified Equation (24) by multiplying it by a coefficient of 0.6. The interception efficiency model can thus be simplified even though it has several limitations, mainly small interception parameter and filter solidity, the latter of which is not too restrictive and can be used for calculations in this work. Several investigators suggested other corrections of Equation (23). For example, Stechkina and Fuchs [61] approximated this relationship by omitting all the terms containing the filter solidity *α* and obtained the following equation:

(26) $$\eta_{R}=\frac{1+R}{2Ku}\left[2\ln(1+R)-1+\frac{1}{{\left(1+R\right)}^{2}}\right]$$

The limitations are the same as for Equation (22) i.e., *R* and *α* must be small. Owing to the omission of filter solidity, the approximation is less accurate with increasing solidity. Therefore, they proposed another modification as follows:

(27) $$\eta_{R}=2.4\alpha^{1/3}R^{1.75}$$

Lee and Gieseke [62] proposed another modification of Equation (24) as follows:

(28) $$\eta_{R}=\frac{1-\alpha }{Ku}\frac{R^{2}}{{\left(1+R\right)}^{\frac{2}{3(1-\alpha )}}}$$

None of the prediction models for interception efficiency (Equations (23)–(28)) considers the gas slip effect. Pich [63] proposed a relationship for interception efficiency, considering gas slip, for small Knudsen numbers:

(29) $$\eta_{R}=\frac{{\left(1+R\right)}^{-1}-(1+R)+2(1+1.996Kn)(1+R)\ln(1+R)}{2(-0.75-0.5\ln\alpha )+1.996Kn(-0.5-\ln\alpha )}$$

Another relationship considering the gas slip effect was developed by Liu and Rubow [54] who further modified the model of Lee and Liu [52] (Equation (25)) by multiplying it by a correction factor for the gas slip as follows:

(30) $$\eta_{R}=0.6\frac{1-\alpha }{Ku}\frac{R^{2}}{1+R}\left(1+\frac{1.996Kn_{f}}{R}\right)$$

#### 2.1.3. SCE Due to Inertial Impaction

Inertial impaction takes place in higher airflow velocities for particles with a larger diameter (mostly larger than 1 µm depending on conditions) due to their higher inertia, which causes them to follow a different trajectory than that of airflow streamlines. The streamlines near the collector abruptly changes. The particle thus separates from the streamlines and hits the collector. Collection efficiency due to inertial impaction depends on Stokes’ number characterizing the particle inertia, which is defined as follows:

(31) $$Stk=\frac{d_{p}^{2}\rho_{p}C_{s}U}{18\mu d_{f}}$$

where *ρ*_p_ is the particle density. If the Stokes’ number is higher than unity, the particles separate from streamlines and hit the collector. On the other hand, for Stokes’ number lower than one, the inertia effect will not take place. Several formulae have been derived for SCE due to inertial impaction. The most often used relationship is that proposed by Stechkina et al. [50]:

(32) $$\eta_{I}=\frac{Stk}{4Ku^{2}}\left(29.6-28\alpha^{0.62}\right)R^{2}-27.5R^{2.8}$$

for 0.0035 < *α* < 0.111 and 0.01 < *R* < 0.4, while for *R* > 0.4, the relationship is modified as follows:

(33) $$\eta_{I}=\frac{Stk}{2Ku^{2}}$$

Landahl and Hermann [64] proposed a relationship based on experimental data for Re_f_ > 10. However, as suggested by Saleh et al. [65], this equation may also be used for Re_f_ < 2. The relationship is as follows:

(34) $$\eta_{I}=\frac{Stk^{3}}{Stk^{3}+0.77Stk^{2}+0.22}$$

Fuchs gave another relationship for impaction efficiency as follows [66]:

(35) $$\eta_{I}=\frac{Stk^{2}}{{\left(Stk+0.25\right)}^{2}}$$

while Gougeon et al. [67] and Friedlander [68] proposed empirical Equations (36) and (37), respectively:

(36) $$\eta_{I}=0.039Stk^{3/2}$$

(37) $$\eta_{I}=0.075Stk^{6/5}$$

Equations (36) and (37) are valid for 0.0263 < Re_f_ < 0.25 and 0.5 < *Stk* < 4.1 and Re_f_ < 1, 0.8 < *Stk* < 2 and *R* < 0.2, respectively. Zhu et al. [69] derived a relationship with no restrictions concerning *Stk*, Re_f_ and *α* as follows:

(38) $$\eta_{I}=\frac{2R(1-\alpha )Stk\sqrt{\alpha }+(1-\alpha )\alpha Stk^{2}}{Ku}$$

Several researchers proposed models accounting for the effect of fiber and particle Reynolds number on the SCE due to inertial impaction. Suneja and Lee [70] derived a relationship for 1 < Re_f_ < 60 and 1 < *Stk* < 20 as follows:

(39) ηI=[1+1.53−0.23lnRef+0.0167(lnRef)2Stk]−2

Ilias and Douglas [71] theoretically investigated inertial aerosol deposition on an isolated cylinder by solving time dependent Navier-Stokes equations. They proposed a correlation for 30 < Re_f_ < 40,000 and 0.07 < *Stk* < 5 as follows:

(40) ηI=Stk3+(1.622×10−4)/Stk1.031Stk3+(1.14+0.04044lnRef)Stk2+0.01479lnRef+0.2013

#### 2.1.4. SCE Due to Adhesion

For adhesion efficiency, several authors proposed empirical relationships for varying material combinations, with different ranges of Reynolds and Stokes numbers. Based on experimental results, an expression for adhesion efficiency was proposed by Ptak and Jaroszczyk as follows [72]:

(41) ηA=190(RepStk)0.68+190

where Re_p_ is the particle Reynolds number calculated as follows:

(42) Repp=dpUρpμ

Re_pp_ is not the standard fluid dynamics Reynolds number, it uses the particle density *ρ*_p_ for the calculation [73]. Equation (41) was accurate for 1 < *Stk* < 120 and 0.4 < Re_f_ < 5.75.

### 2.2. Efficiency Prediction of CPM

**Figure 3:**
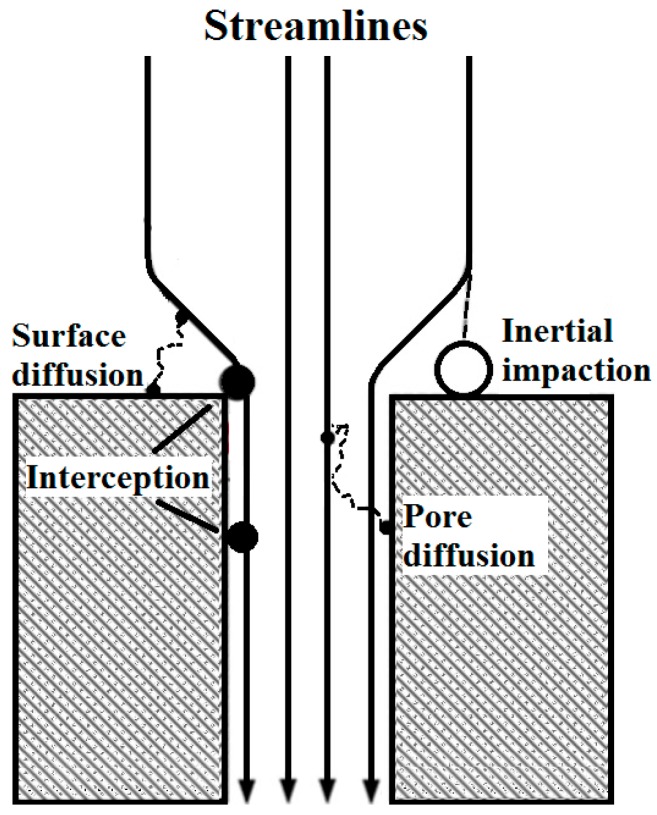
Schematic filtration mechanisms involved in separation on a CPM.

CPMs are thin polycarbonate membranes with circular pores. The theoretical prediction of the filtration efficiency is based on several mechanisms similar to fibrous filters but with physically different meanings (Figure 3).

The theoretical impaction efficiency *η*_I_ for Nuclepore filters can be calculated using the model proposed by Pich [74] as follows:

(43) $$\eta_{I}=\frac{2{\eta^{\prime }}_{I}}{1+\xi }-\frac{{\eta^{\prime }}_{I}{}^{2}}{{\left(1+\xi \right)}^{2}}$$

where ${\eta^{\prime }}_{I}$ and ξ are calculated as follows:

(44) $${\eta^{\prime }}_{I}=2Stk\sqrt{\xi }+25Stk^{2}\exp\left(-\frac{1}{Stk\sqrt{\xi }}\right)-2Stk^{2}\xi$$

(45) $$\xi =\frac{\sqrt{P}}{1-\sqrt{P}}$$

where *P* is the membrane porosity and *Stk* is the Stokes number and is calculated as follows:

(46) $$Stk=\frac{d_{p}^{2}\rho_{p}C_{c}U}{9\mu d_{o}}$$

with *C*_c_ as the slip correction factor and calculated as follows [66]:

(47) $$C_{c}=1+2.49\frac{\lambda }{d_{p}}+0.84\frac{\lambda }{d_{p}}\exp\left(-0.44\frac{d_{p}}{\lambda }\right)$$

The diffusion efficiency in pores *η*_D_ can be calculated as follows [75]:

(48) $$\eta_{D}=2.56N_{D}^{2/3}-1.2N_{D}-0.177N_{D}^{4/3}$$

if *N*_D_ < 0.01 or

(49) $$\begin{array}{l} \eta_{D}=1-0.819\exp\left(-3.657N_{D}\right)-0.098\exp\left(-22.305N_{D}\right)\\ -0.032\exp\left(-56.95N_{D}\right)-0.016\exp\left(-107.6N_{D}\right) \end{array}$$

if *N*_D_ > 0.01, where *N*_D_ is:

(50) $$N_{D}=\frac{4ZPD}{d_{o}^{2}U}$$

where *D* is diffusion coefficient calculated according to Equation (5) and *d*_o_ is the pore diameter. The interception efficiency on pore opening *η*_R_ can be calculated using the model suggested by Spurny et al. [75]:

(51) $$\eta_{R}=R_{o}\left(2-R_{o}\right)$$

where *R*_o_ is the interception parameter for capillary pore filters calculated as follows:

(52) $$R_{o}=\frac{d_{p}}{d_{o}}$$

Nanoparticles can also deposit on the front surface of Nuclepore filters when particles are smaller than 100 nm and face velocity is low. The surface-diffusion efficiency *η*_DS_ can be calculated using the expression proposed by Manton [76]:

(53) $$\eta_{\mathrm{DS}}=1-\exp\left[\frac{-\beta_{1}\delta^{2/3}}{1+\left(\beta_{1}/\beta_{2}\right)\delta^{7/15}}\right]$$

where *β*_2_ = 4.5 and *β*_1_ and *δ* are coefficients that are calculated as follows:

(54) $$\beta_{1}=4.57-6.46P+4.58P^{2}$$

(55) $$\delta =\frac{2D\sqrt{P}}{d_{o}U}$$

The total efficiency *η* is calculated as follows:

(56) $$\eta =1-(1-\eta_{I})(1-\eta_{D})(1-\eta_{R})(1-\eta_{\mathrm{DS}})$$

## 3. Materials and Methods

### Hollow-Fiber Membranes

**Figure 4:**
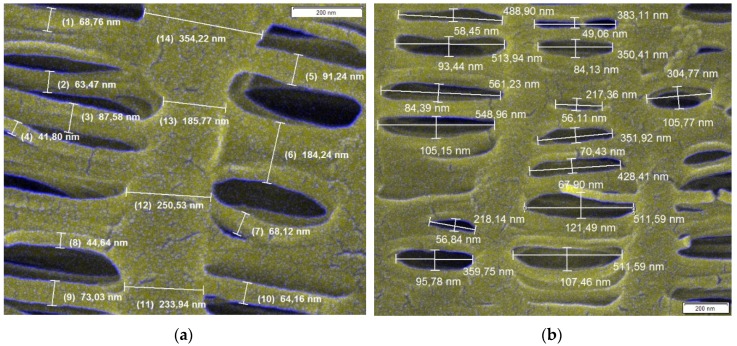
Evaluation of collector diameter (**a**) and pore size (**b**) from SEM images using Stream Motion software.

HFM is a special type of membrane geometry. HFM modules are characterized by compactness as they contain a high filtration area within a small volume. Figure 4 shows a HFM pore structure. As mentioned above, two different approaches were chosen. One is based on SCE (Figure 4a) and the other based on a capillary pore approach using models developed for predicting the efficiency of Nuclepore filters considering pore dimensions (Figure 4b). Based on the SEM images, dimensions of individual collectors (Figure 4a) and pores (Figure 4b) were determined using Stream Motion software (Olympus Corporation, Shinjuku, Japan). Using these dimensions, a weighted average size of collector/pore *d*_f(o)_ was calculated as follows:

(57) $$d_{f(o)}=\frac{\sum_{i=1}^{N}n_{i}d_{f(o)i}}{N}$$

where *d*_f(o)*i*_ is an individual collector (pore) size, *n_i_* is the number of collectors/pores with a given size *d*_f(o)*i*_ and *N* is the number of all measured collectors/pores, i.e., number of measurements obtained from the SEM pictures. The average collector/pore size is thus a weighted average of 125 values. The weighted average pore size was calculated using pore dimensions of the elliptical shape (the major and minor axes). The largest particle able to penetrate through the membrane is mostly given by the smaller pore dimension (i.e., that of minor axe). However, due to the random motion and shape of particles, some particles larger than the minor axe length can penetrate through the membrane. Therefore, the weighted average was calculated using both axes’ dimensions, giving a larger average pore size. This step ensures that the results of the predicted efficiencies will not be overrated. The main parameters of the membrane structure and conditions for which the models were compared are shown in Table 1. For the model comparison, we also used the standard deviation of pore and collector average diameter to depict uncertainty bounds. For the sake of brevity, this was done for final results only, i.e., overall efficiency.

## 4. Results and Discussion

In this section, results obtained using different models are compared when applied on the parameters of the HFM pores structure. Two different approaches were used as mentioned in the previous section, i.e., the approach based on models developed for fibrous filters and a model for membrane filters.

### 4.1. Fibrous Filters

**Figure 5:**
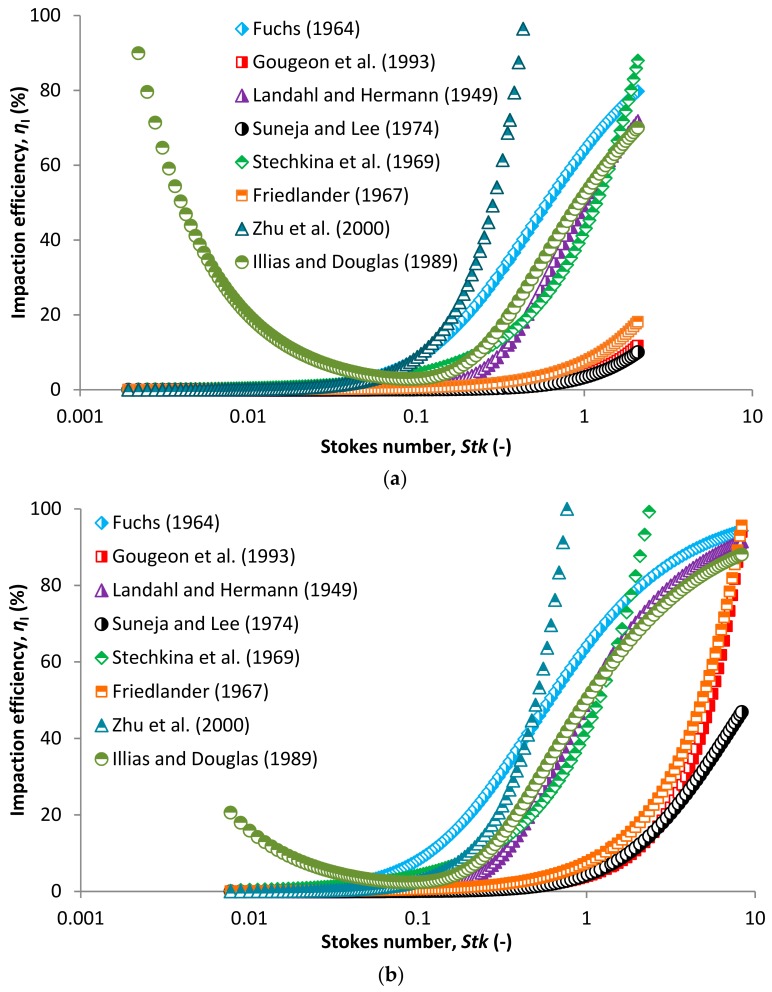
Comparison of impaction efficiency based on different models and airflow velocity of 5 cm/s (**a**) and 20 cm/s (**b**) in relation to Stokes number.

Figure 5 shows SCE due to the inertial impaction based on different models for a face velocity of 5 cm/s (a) and 20 cm/s (b) in relation to *Stk*. Stokes number is a governing parameter of inertial impaction mechanism based on which one can decide if the inertial impaction mechanism dominates at conditions adopted in a filtration process. Moreover, the use of *Stk* is more appropriate compared to the relation of efficiency to particle diameter. *Stk* relates to the particle diameter itself, particle density, collector diameter, face velocity and other parameters governing the mechanisms taking place during aerosol filtration (Equation (31)). Inertial impaction significantly governs the separation if *Stk* > 1, which is mostly true for particles larger than 1 µm at higher face velocities. At such conditions, the particles have higher inertia and easily separate from airflow streamlines and hit the collector (Figure 2). This is true for higher face velocities even for particles smaller than 1 µm. However, for a lower face velocity of 5 cm/s (Figure 5a) we can see that impaction efficiency starts to increase significantly for *Stk* above 0.1. This is caused by the ultrafine collectors in the membrane structure (Figure 4a). The average collector diameter is 90 nm, giving a higher *Stk* even for smaller particles as *Stk* is inversely proportional to collector diameter according to Equation (31), thus giving higher theoretical efficiencies compared to general fibrous filters. The membrane structure is more similar to nanofiber based filters, though it is more dense (Figure 4, compare e.g., [19,37,77,78,79]). Different impaction efficiency models in relation to particle diameter are compared by face velocity in supplementary material (Figure S1). The courses of impaction efficiency in relation to particle diameter are practically the same as those in relation to *Stk* and are shown in supplementary material (Figure S2). The only model, which significantly deviates from the others is that derived by Ilias and Douglas [71] which predict high impaction efficiency at low *Stk*. This relationship is valid for 0.07 < *Stk* < 5. The bottom limit of *Stk* is the point where the curve increases in the direction of decreasing *Stk*, so it is necessary to omit this part of the curve because it is clear that the impaction regime in this *Stk* region does not occur.

**Figure 6:**
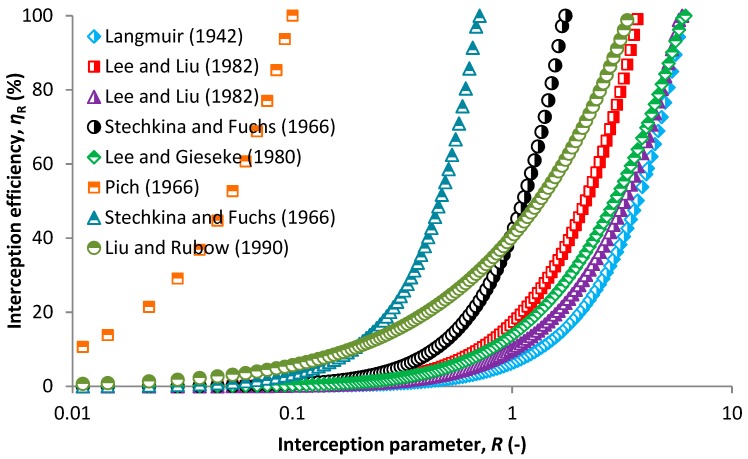
SCE due to interception based on various models in relation to interception parameter.

Figure 6 shows a single fiber efficiency due to the interception mechanism in relation to interception parameter. Interception may play an important role in nanoparticle filtration if the collector diameters are small [80] and starts to dominate at an interception parameter of 0.1 [81]. This is true for most of the models except for that derived by Pich [63] (Equation (29)) which predicts high interception efficiencies also for very small interception parameters under 0.1 corresponding to particle sizes smaller than 10 nm (Supplementary Figure S3). The Equation (29) was derived for small fiber Knudsen numbers which is not fulfilled for the given collector diameter. Therefore, the Knudsen number is higher and the model overrates the results to lower particle sizes. Liu and Rubow [54] derived another relationship (Equation (30)) considering the gas slip effect, which is more appropriate for very small collector diameters. The interception mechanism is independent of face velocity, which is the main difference from inertial impaction and Brownian motion. This is, however, not true for model of Langmuir [60] where the interception efficiency also dependent on the fiber Reynolds number which is given by the face velocity (Figure S3a).

**Figure 7:**
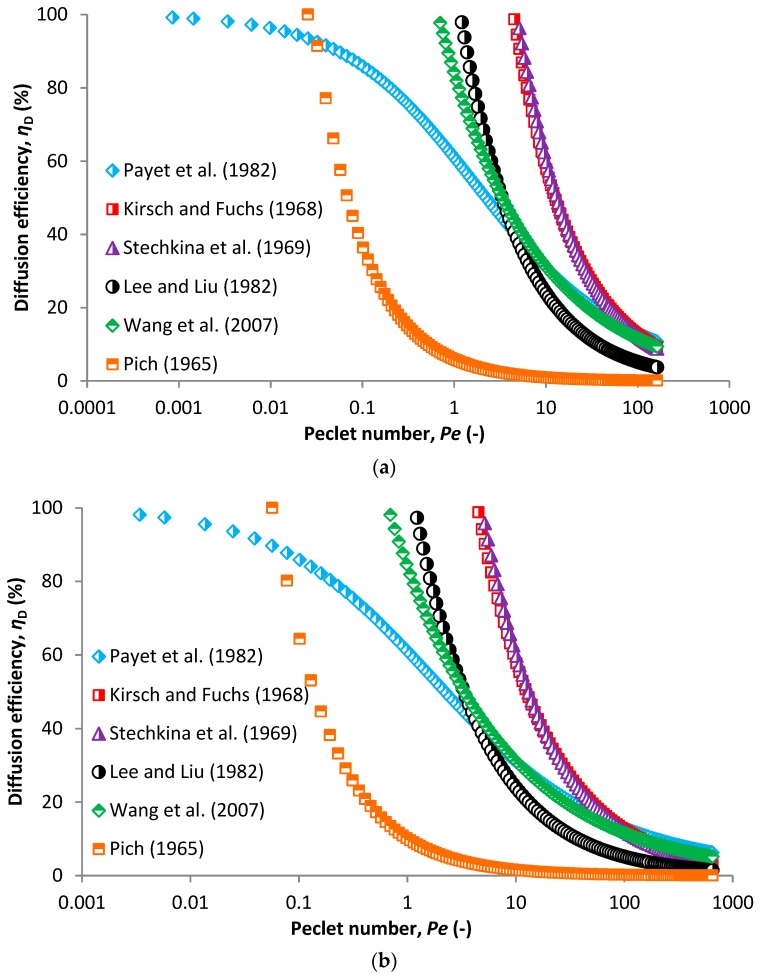
Comparison of SCE due to diffusion mechanisms based on different models for an airflow velocity of 5 cm/s (**a**) and 20 cm/s (**b**) in relation to the Peclet number.

Brownian motion (diffusion) is another important mechanism occurring when separating particles from air. Unlike for inertial impaction, this mechanism is enhanced at very small face velocities and for very small particles that are mostly under 100 nm in size. The governing parameter for Brownian motion is the Peclet number, which is a ratio of convection to diffusion transport rate. SCE due to diffusion increases with a decreasing Peclet number, i.e., decreasing particle size (Figure 7). With increasing airflow velocity, the Peclet number is shifted to higher values which diminishes the capturing effect caused by random motions of particles (compare Figure 7a,b). Therefore, with increasing velocity, the SCE due to diffusion decreases and is shifted to lower particle sizes. Comparison of efficiency/particle size curves by face velocity calculated using different models are shown in supplementary material in Figure S4, a comparison of individual models is in Figure S5. The most appropriate model for SCE due to diffusion is Equation (18). This model developed by Payet et al. [55] covers even very small particles for which the other models give an efficiency that is higher than 1 (Figure 7).

**Figure 8:**
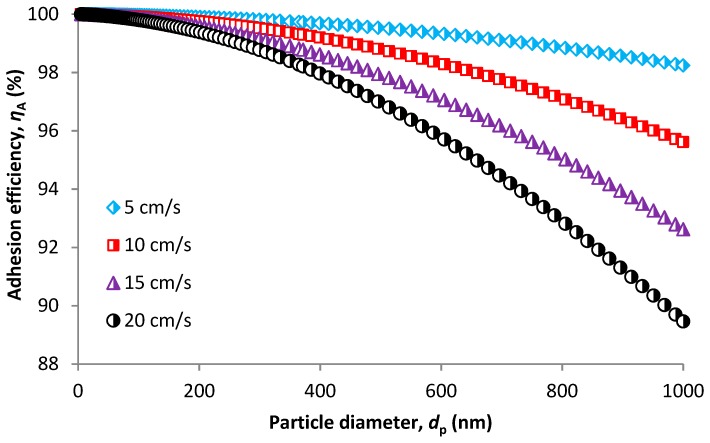
Collection efficiency due to the adhesion effect.

Figure 8 shows adhesion efficiency in relation to particle size based on the model developed by Ptak and Jaroszczyk [72] (Equation (41)). This mechanism is not often considered in theoretical predictions. However, we also use this model to completely describe the mechanical capture of particles in which adhesion plays an important role due to re-entrain and rebound effects. We also calculated single fiber efficiency according to Equation (3), which is the product of collision efficiency (a sum of SCE due to impaction interception and diffusion) and adhesion efficiency presented by the values predicted using Equation (41). Adhesion efficiency is mostly higher for smaller particles and lower face velocities as shown in Figure 8. This is given by adhesion energy between a particle and a fiber as follows:

(58) $$E=\frac{Hd_{p}}{6a_{0}^{2}}$$

where *H* is the Hamaker constant and *a*_0_ is the adhesion distance. Adhesion energy is directly proportional to the particle size, therefore, higher energy is necessary to keep a larger particle attached to the fiber. It is similar for face velocity, which is mostly assumed the same as the impact velocity of the particle colliding with the fiber surface. The impact velocity should be less than the critical velocity *ν* derived from the adhesion energy given as follows [46]:

(59) $$v<\sqrt{\frac{H}{4\pi \rho a_{0}d_{p}^{2}}}$$

The Hamaker constant can be calculated as follows [46,82]:

(60) $$H=\frac{3}{4}k_{B}T\left(\frac{\varepsilon_{1}-\varepsilon_{3}}{\varepsilon_{1}+\varepsilon_{3}}\right)\left(\frac{\varepsilon_{2}-\varepsilon_{3}}{\varepsilon_{2}+\varepsilon_{3}}\right)+\frac{3h\vartheta_{e}}{8\sqrt{2}}\frac{\left(n_{1}^{2}-n_{3}^{2}\right)\left(n_{2}^{2}-n_{3}^{2}\right)}{\sqrt{\left(n_{1}^{2}+n_{3}^{2}\right)\left(n_{2}^{2}+n_{3}^{2}\right)\left[\sqrt{\left(n_{1}^{2}+n_{3}^{2}\right)}+\sqrt{\left(n_{2}^{2}+n_{3}^{2}\right)}\right]}}$$

where *ε* is the static dielectric constant, *n* is the refractive index, *h* is the Planck constant and *ϑ_e_* is the main electronic absorption frequency typically around 3 × 10^15^ s^−1^. The subscript notation 1, 2 and 3 of *ε* and *n* indicate the particle, membrane surface and fluid, respectively. The typical value of Hamaker constant ranges between 10^−19^ and 10^−20^ [83]. However, significant influence will also have particle surface charges, which can cause the membrane to act as an electret filter, so the particles may be captured due to electrostatic forces. In this work however, we focus on the mechanical means of filtration only, so this effect is not considered.

#### Overall SCE and overall Filtration Efficiency

**Figure 9:**
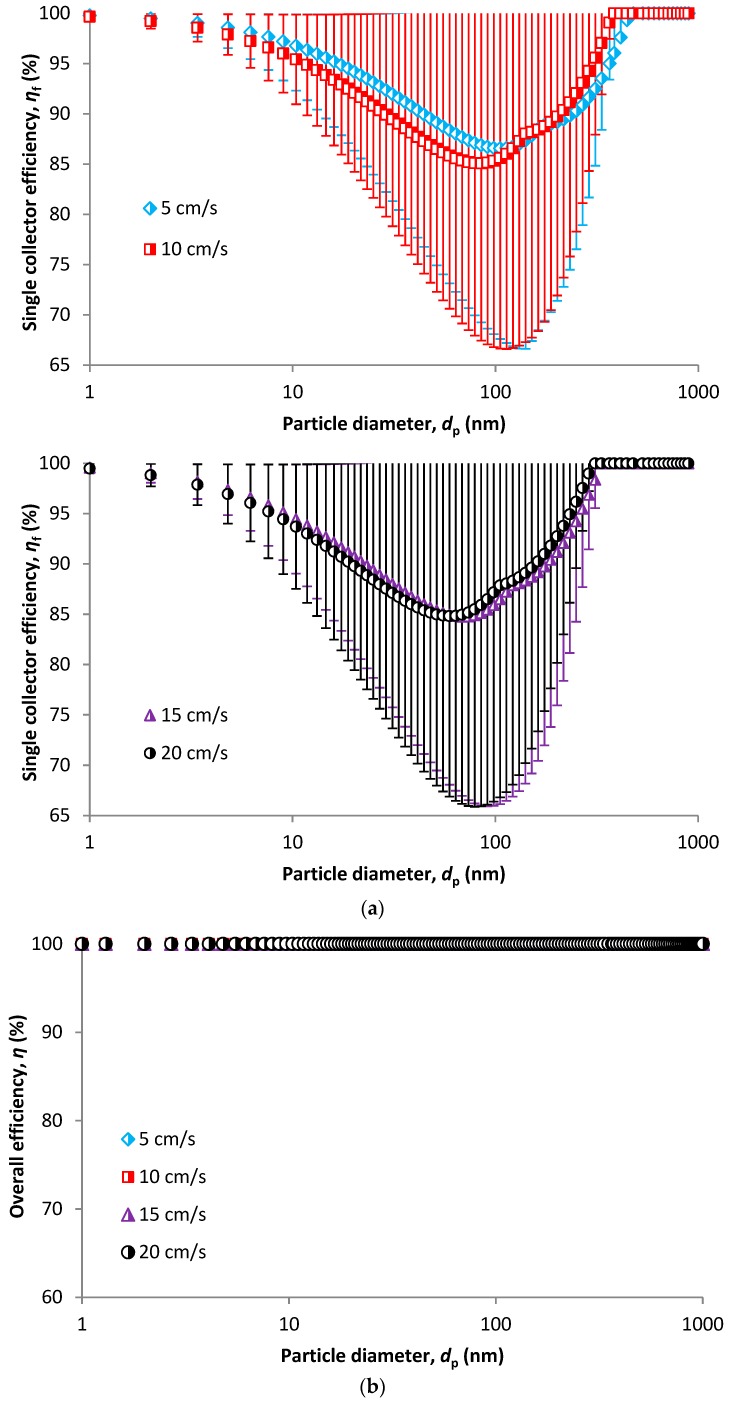

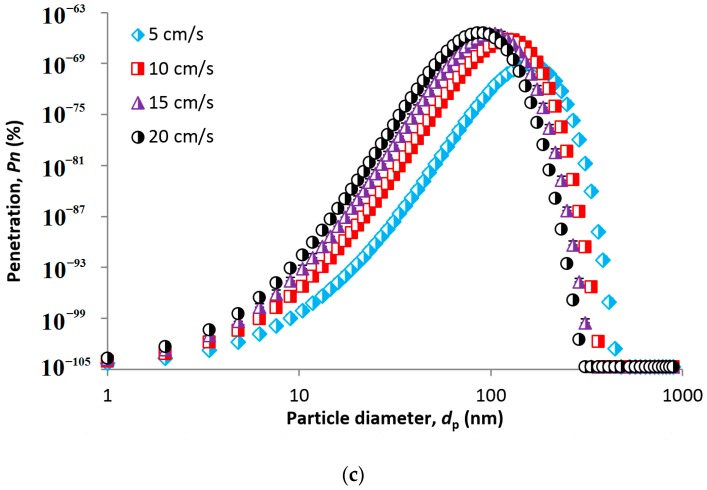
Single collector efficiency (**a**), overall filter efficiency (**b**) and overall penetration (**c**).

Overall SCE is shown in Figure 9a. This is a typical shape of efficiency/particle size curve with a minimum corresponding to most penetrating particle size (MPPS). The left-hand side of the minimum is governed by the diffusion mechanism while interception and inertial impaction are responsible for the right-hand side. However, the curves in Figure 9a correspond only to one single filter fiber, i.e., one collector of the HFM structure (Figure 4a). To get an overall membrane efficiency, it is necessary to recalculate the SCE to a whole membrane structure using Equation (2). The results are shown in Figure 9b,c. After recalculating, we get 100% removal efficiency for all particle sizes (Figure 9b). Figure 9c shows the same expressed as penetration, i.e., the amount of particles which can penetrate through the membrane, which is in order of 10^−66^ which is practically equaled to zero. The results shown in Figure 9a are single collector efficiencies calculated using models for diffusion (Equation (18)), interception (Equation (21)), impaction (Equation (35)) and adhesion (Equation (41)). So it is an example of one selected combination of models for individual mechanism. The other was not calculated as it was assumed that the result would be the same or would vary somewhere in the order of 10^−70^, which is negligible.

The main reason for these results is the high solidity of the HFM structure, which is 0.48, while most of the fibrous filters have solidity between 0.01–0.3 [44] and most of the models are developed for this solidity range. Moreover, the membrane collector diameter is very small, giving a very dense structure. If we look at Figure 4a, we can see collector diameters of about 100 nm in size. The thickness of the membrane wall is 36 µm. This means that there are about 360 such layers in the membrane wall creating a dense network that is very hard for particles to penetrate. Therefore, the results seems to be reasonable. In practice, this membrane could serve as an absolute filter which are used for aerosols which must have 100% removal efficiency. Such aerosols include some radioactive particles, toxic aerosols and viruses.

### 4.2. CPM

**Figure 10:**
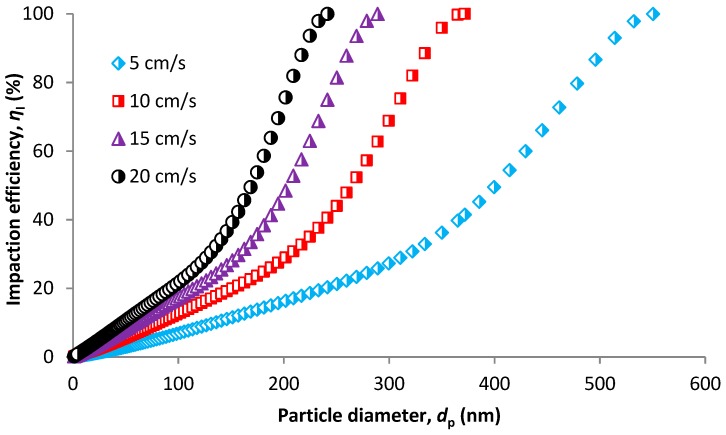
Impaction efficiency based on CPM model.

The approach based on membrane pore size instead of membrane fiber diameter is presented in this section. Inertial impaction is stronger for larger particles at higher velocities, which is in accordance with theory. However, the model of Pich [74] is less accurate as it does not consider the possible sieving effect in membrane filters i.e., complete capture of particles on the membrane surface for particles larger than membrane pore size. This is obvious from Figure 10. The membrane pore size considered in the calculations is 205 nm (Table 1). If circular pores are assumed, which is a simplification in the model, we should obtain 100% efficiency for particles above 205 nm regardless of the face velocity. This is not seen to be true from Figure 10.

**Figure 11:**
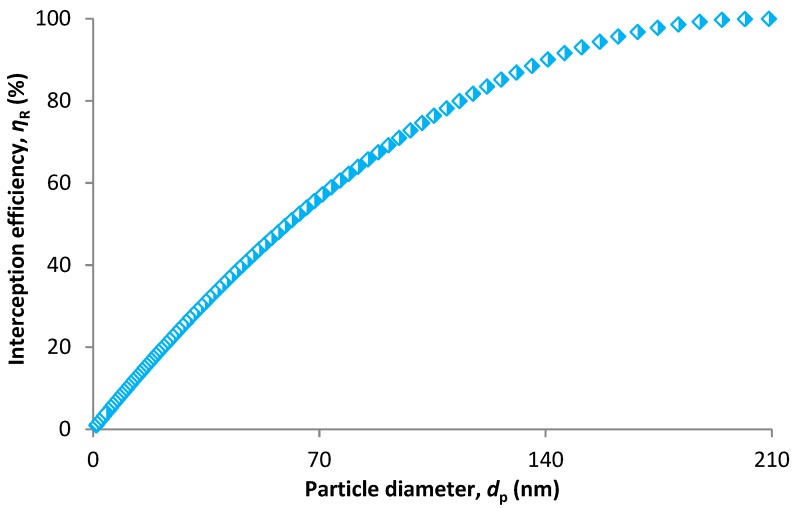
Interception efficiency based on CPM model.

More plausible results are obvious for interception efficiency (Figure 11). The interception efficiency increases up to a particle size of 202 nm with an efficiency of 99.97%. For a particle size of 209 nm (slightly larger than pore size), efficiency is 100% which is reasonable. Therefore, the model proposed by Spurny et al. [75] (Equation (51)) seems to be accurate for the structure of polypropylene HFMs.

**Figure 12:**
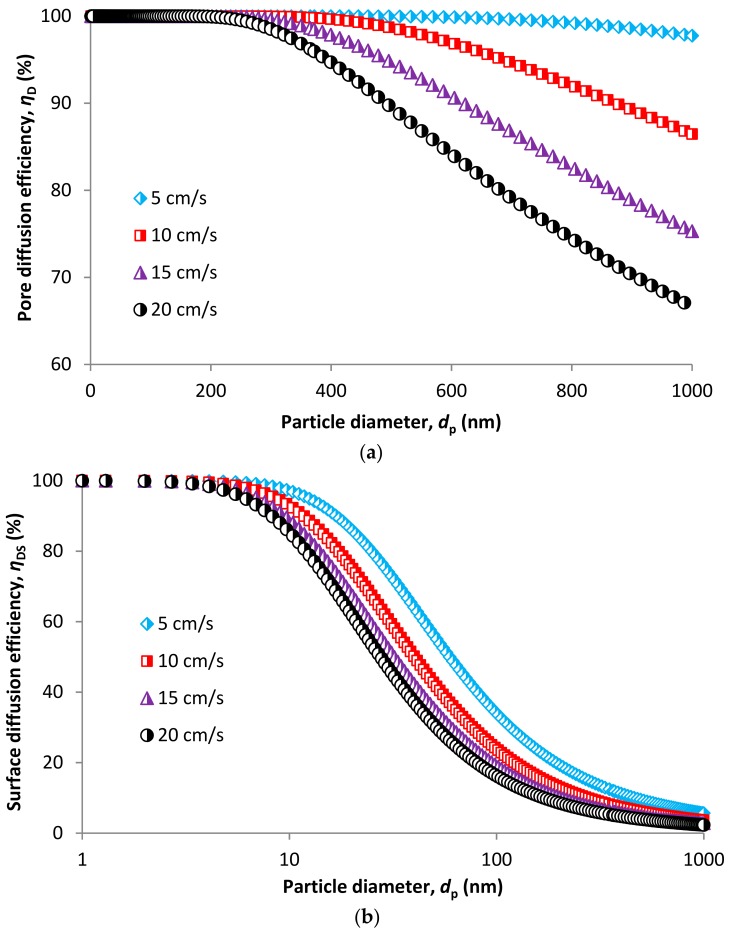
Collection efficiency due to diffusion in pores (**a**) and on the membrane surface (**b**).

Diffusion is an important part of the overall efficiency. We can distinguish between diffusion capture in pores and diffusion capture on membrane surface (Figure 3). Prediction models were developed for both (Equations (48) and (53)). Figure 12a shows pore diffusion efficiency. To talk about diffusion capture within membrane pore structure is possible only for particles smaller than the largest pore size (i.e., smaller than 205 nm). Larger particles will only be a subject to surface diffusion capture (Figure 12b) which is possible for whole particle size range. From Figure 12a, a similar problem for the model for impaction efficiency is obvious. While efficiency for impaction should be 100% for particles above 205 nm, pore diffusion should be equaled to zero because no particle larger than 205 nm cannot penetrate the pore structure, so there is no diffusion capture of these particles.

**Figure 13:**
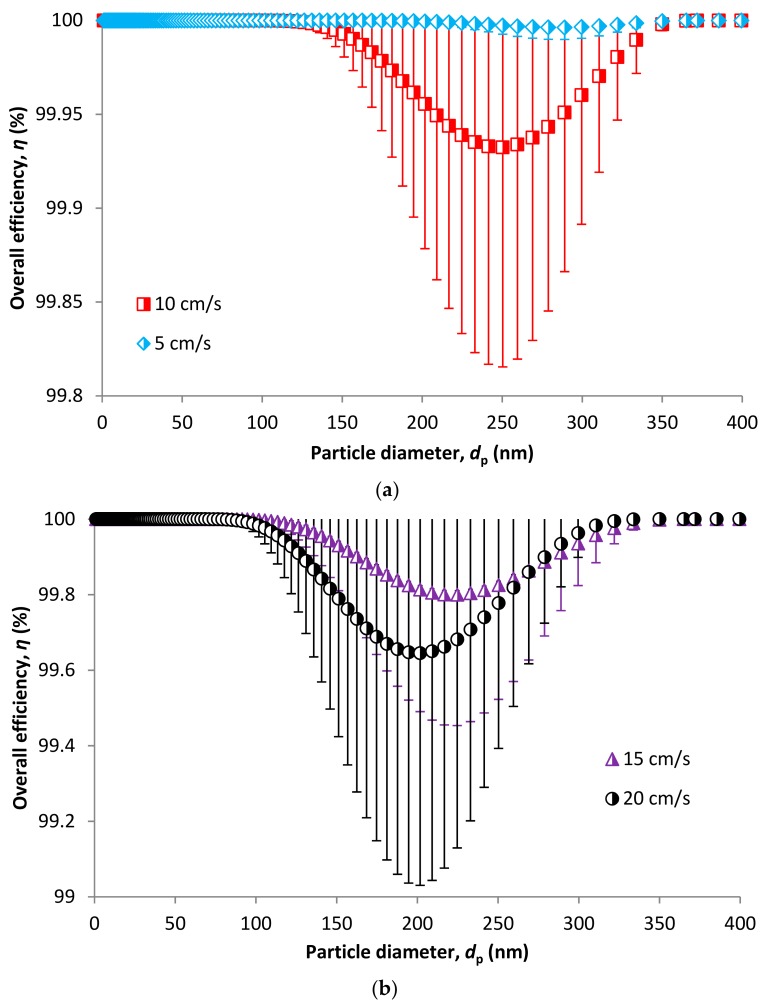
Overall efficiency in relation to particle size based on CPM model for a velocity of 5 and 10 cm/s (**a**) and 15 and 20 cm/s (**b**).

Overall efficiency is predicted based on the models for individual mechanisms and calculated using Equation (56). Figure 13 shows 99.997% MPPS (290 nm) efficiency at a velocity of 5 cm/s. With increasing velocity, the efficiency for MPPS decreases. However, it is still in the range of 99.7% at a velocity of 20 cm/s. MPPS is shifted to smaller particle size with velocity. It is 250, 225 and 202 nm for 10, 15 and 20 cm/s, respectively. This model gives more realistic results compared to the model for fibrous filters, where unconditional 100% efficiency was obtained for all face velocities.

## 5. Conclusions

Prediction models for air filtration efficiency of fibrous and membrane filters were numerically compared by applying an HFM pore structure. With some assumptions, these models can be used for predictions of the aerosol separation efficiency of HFMs. Fibrous filter models give 100% efficiency no matter what level of face velocity, i.e., zero penetration. This is given by very small collectors in membrane structure similarly to nanofibrous filters. Compared to nanofibrous filters, HFMs have a very high solidity of 0.48. The HFM structure is very dense and the calculations can overestimate, as most of the models predict filter efficiency for solidity up to 0.3. CPM models predict efficiencies that are more realistic. Penetration up to 0.00014% was calculated for a face velocity of 20 cm/s. CPM models seem to give more plausible results for these HFMs, however, an experimental verification should be appropriate to compare accuracy of both approaches. However, this is rather a suggestion for another study, as this verification would probably be challenging, concerning experimental work. Thus, it would also be possible to empirically develop a new accurate model for HFMs.

## Figures and Tables

**Table 1:** Parameters of HFM pore structure and conditions used for calculations.

Fiber wall thickness, *Z* (µm)	36
Average pore size, *d*_o_ (nm)	205 ± 157
Average collector diameter, *d*_f_ (nm)	90 ± 83
Solidity, α (%)	48
Porosity, *ε* (%)	52
Temperature, *T* (K)	296.15
Air density, *ρ* (kg m^−3^)	1.21
Air dynamic viscosity, *µ* (Pa s)	1.83 × 10^−5^
Particle density, *ρ*_p_ (kg m^−3^)	1060
Mean free path of air molecules, *λ* (nm)	67.3

## Supplementary Materials

The following are available online at http://www.mdpi.com/2079-4991/8/6/447/s1, Figure S1: SCE due to inertial impaction based on model of Stechkina et al. (a), Landahl and Herman (b), Fuchs (c), Gougeon et al. (d), Suneja and Lee (e), Friedlander (f), Zhu et al. (g) and Illias and Douglas (h), Figure S2: Comparison of impaction efficiency based on different models and airflow velocity of 5 cm/s (a) and 20 cm/s (b), Figure S3: Collection efficiency due to interception mechanism based on Langmuir model for different airflow velocities (a) and a comparison of SCE due to interception based on models developed by various researchers (b), Figure S4: SCE due to diffusion mechanism based on mathematical models developed by Payet et al. (a), Kirsch and Fuchs (b), Stechkina et al. (c), Lee and Liu (d), Wang et al. (e) and Pich (f), Figure S5; Comparison of SCE due to diffusion mechanism based on different models for an airflow velocity of 5 cm/s (a) and 20 cm/s (b).
