# Supplementary material for: Numerical Comparison of Prediction Models for Aerosol Filtration Efficiency Applied on a Hollow-Fiber Membrane Pore Structure
Source: Nanomaterials (Basel). 2018 Jun 19;8(6):447. doi: 10.3390/nano8060447 (PMC6027286; doi:10.3390/nano8060447)
Supplement: Supplementary file 1 [file nanomaterials-08-00447-s001.pdf]

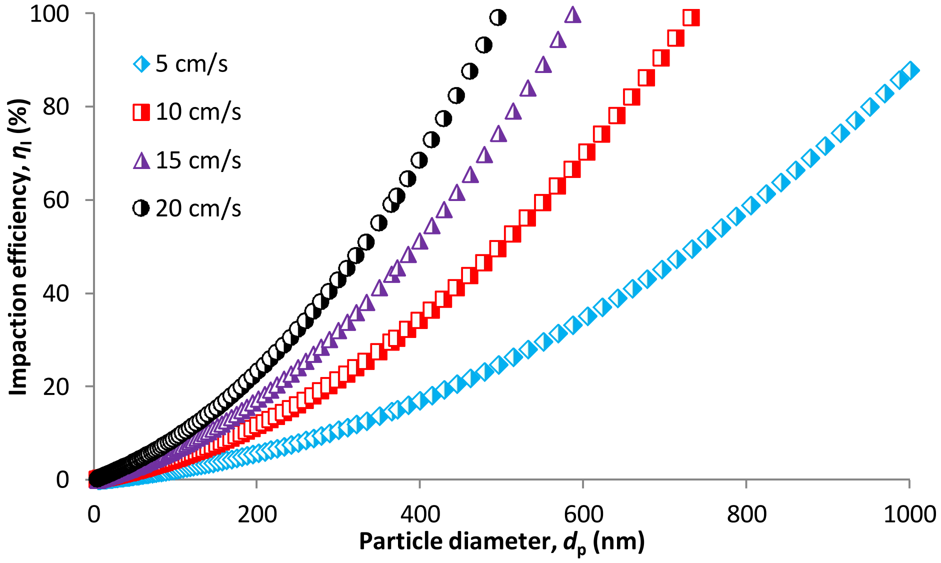

Impaction efficiency, $\eta_I$ (%)
100
80
60
40
20
0
0
200
400
600
800
1000
Particle diameter, $d_p$ (nm)
5 cm/s
10 cm/s
15 cm/s
20 cm/s


(a)

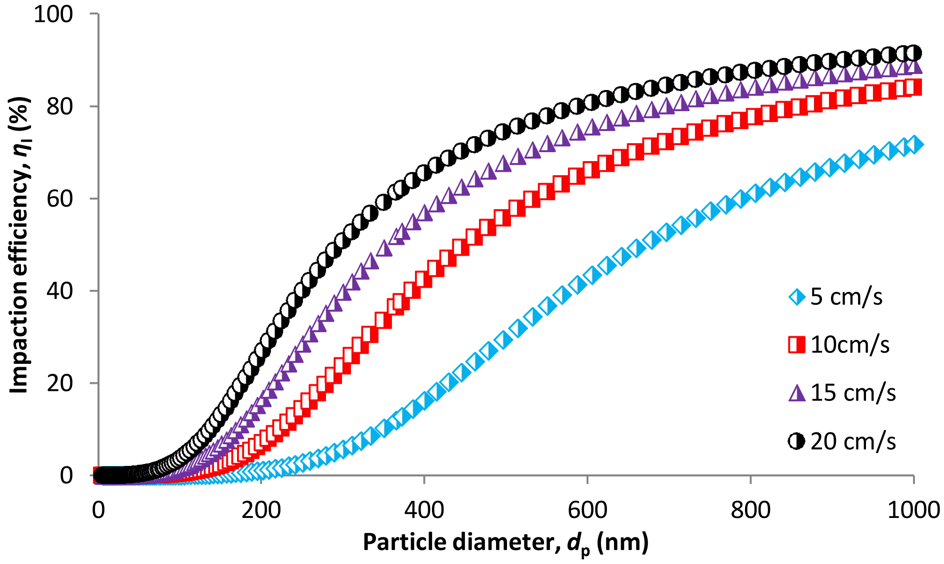

Impaction efficiency, $\eta_I$ (%)
100
80
60
40
20
0
0
200
400
600
800
1000
Particle diameter, $d_p$ (nm)
5 cm/s
10cm/s
15 cm/s
20 cm/s


(b)

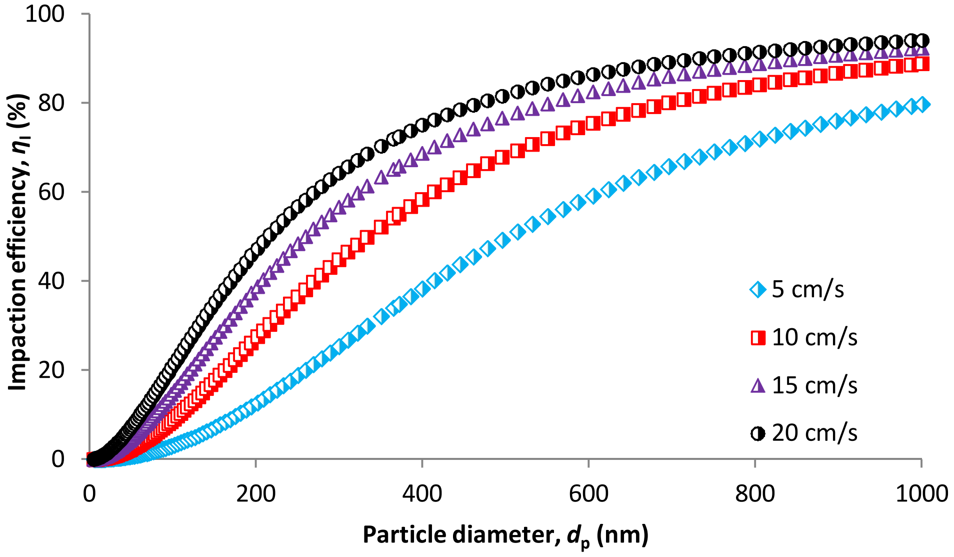

Impaction efficiency, $\eta_I$ (%)
100
80
60
40
20
0
0
200
400
600
800
1000
Particle diameter, $d_p$ (nm)
5 cm/s
10 cm/s
15 cm/s
20 cm/s


(c)

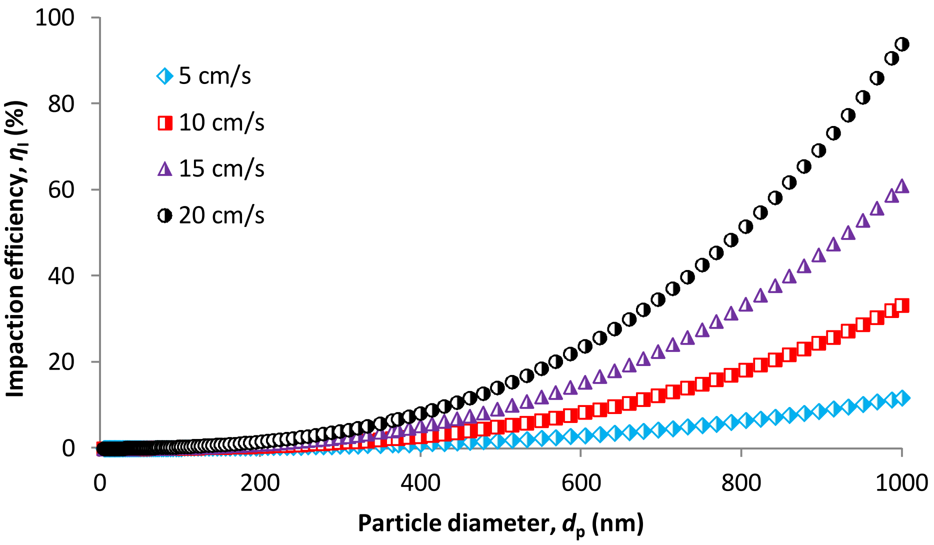
Impaction efficiency, $\eta_I$ (%)
Particle diameter, $d_p$ (nm)
5 cm/s
10 cm/s
15 cm/s
20 cm/s
0
20
40
60
80
100
0
200
400
600
800
1000

(d)

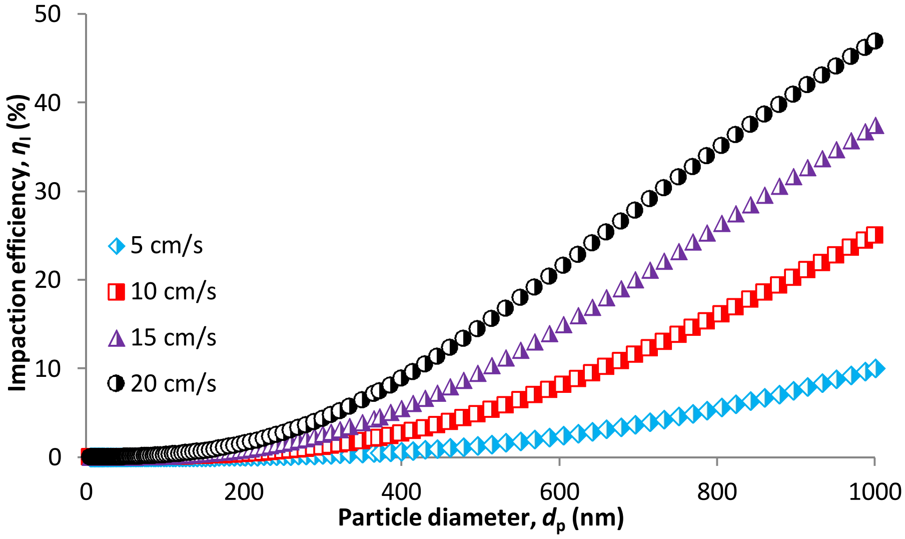
Impaction efficiency, $\eta_I$ (%)
Particle diameter, $d_p$ (nm)
5 cm/s
10 cm/s
15 cm/s
20 cm/s
0
10
20
30
40
50
0
200
400
600
800
1000

(e)

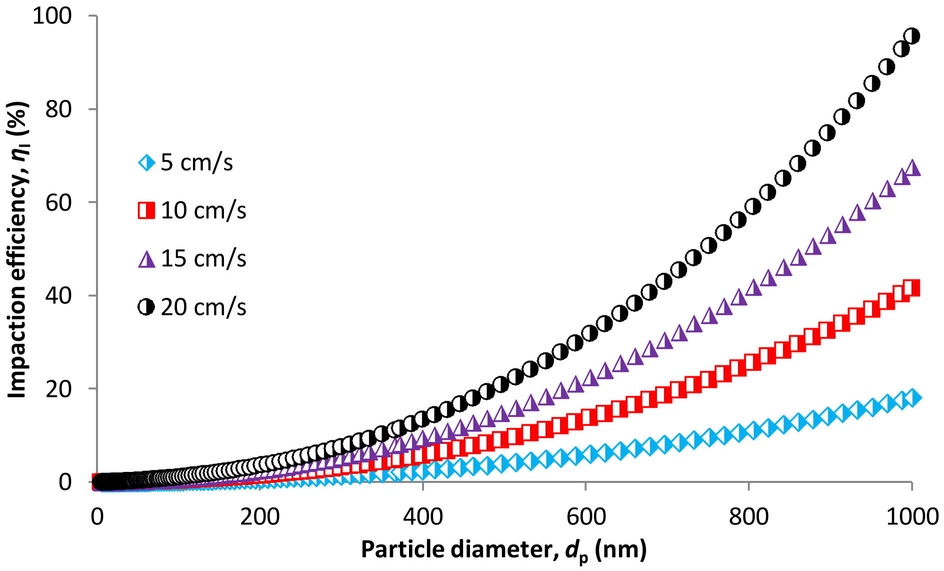
Impaction efficiency, $\eta_I$ (%)
Particle diameter, $d_p$ (nm)
5 cm/s
10 cm/s
15 cm/s
20 cm/s
0
20
40
60
80
100
0
200
400
600
800
1000

(f)

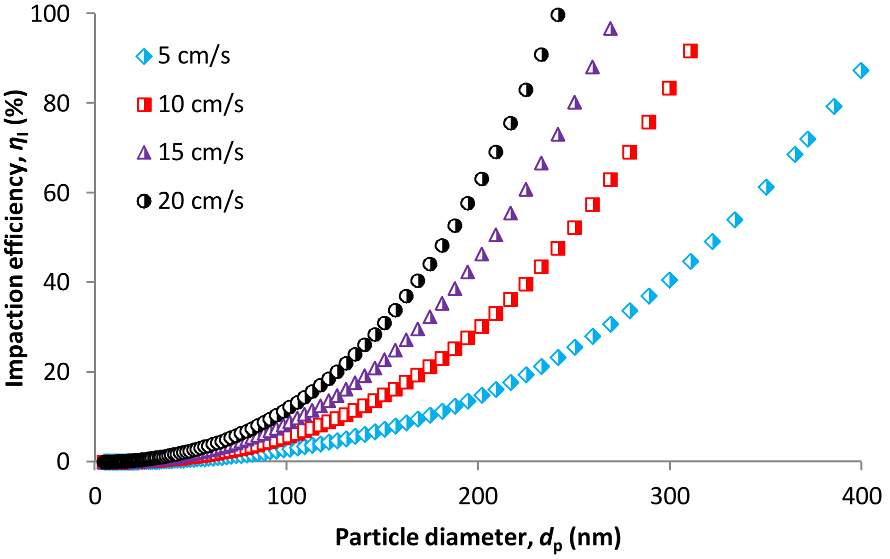


**(g)**

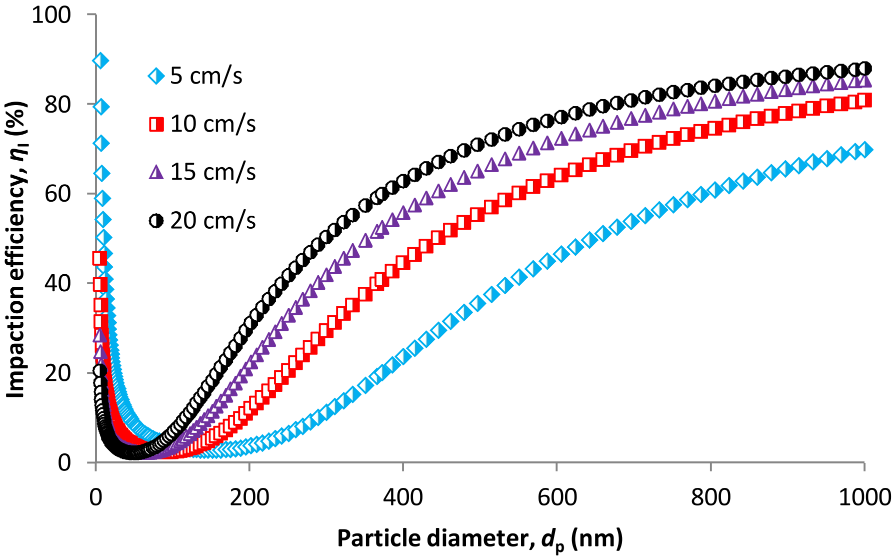


**(h)**

**Figure S1.** SCE due to inertial impaction based on model of Stechkina et al. (a), Landahl and Herman (b), Fuchs (c), Gougeon et al. (d), Suneja and Lee (e), Friedlander (f), Zhu et al. (g) and Illias and Douglas (h)

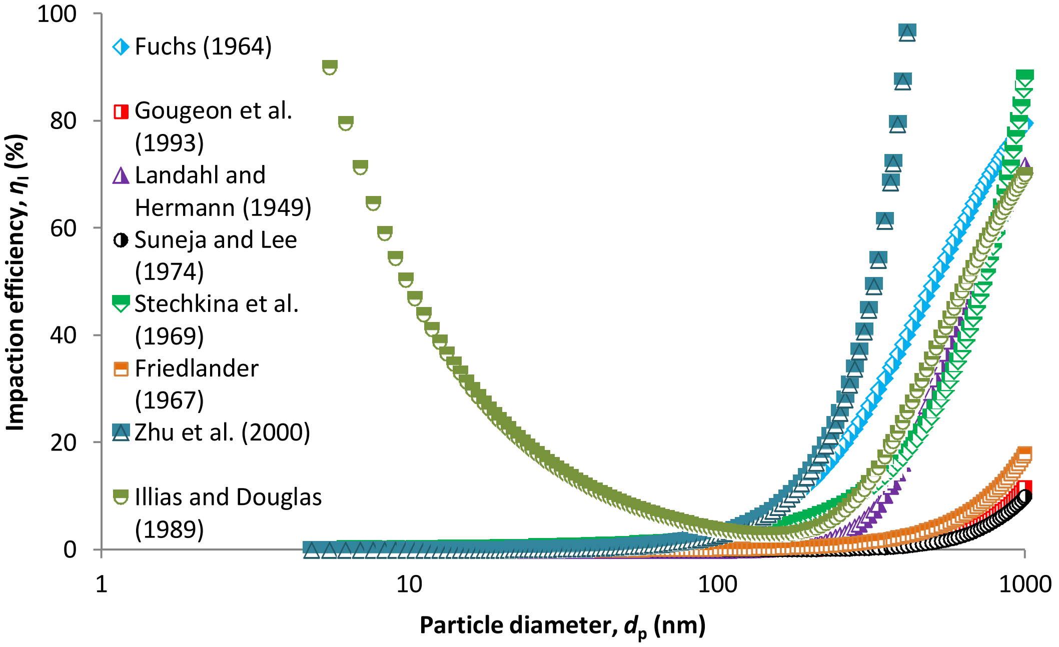


**a)**

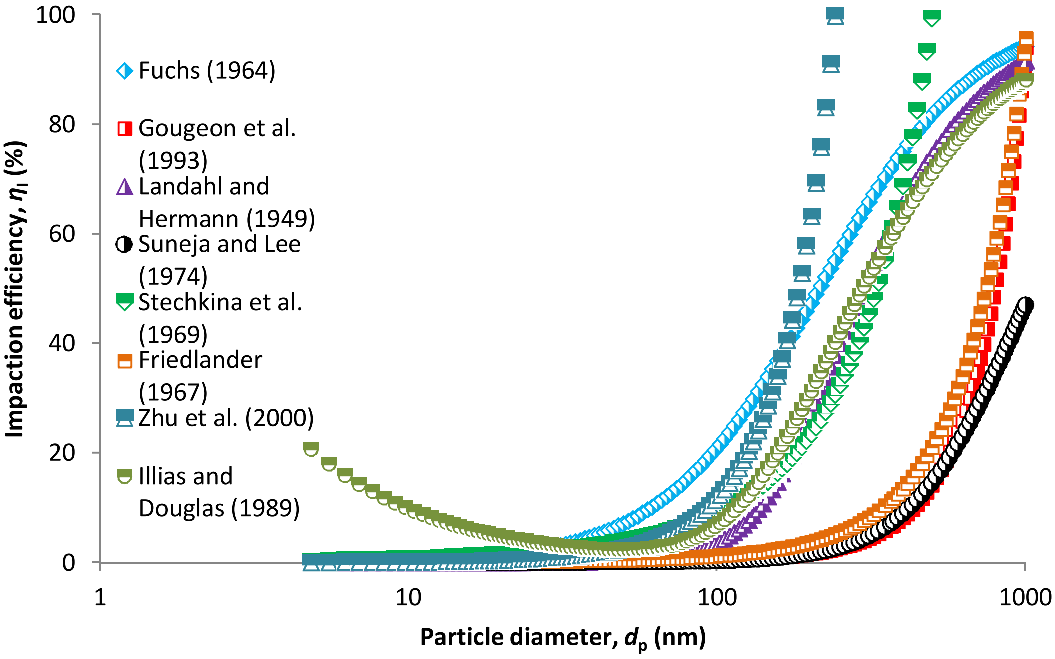


**(b)**

**Figure S2.** Comparison of impaction efficiency based on different models and airflow velocity of 5 cm/s (a) and 20 cm/s (b)

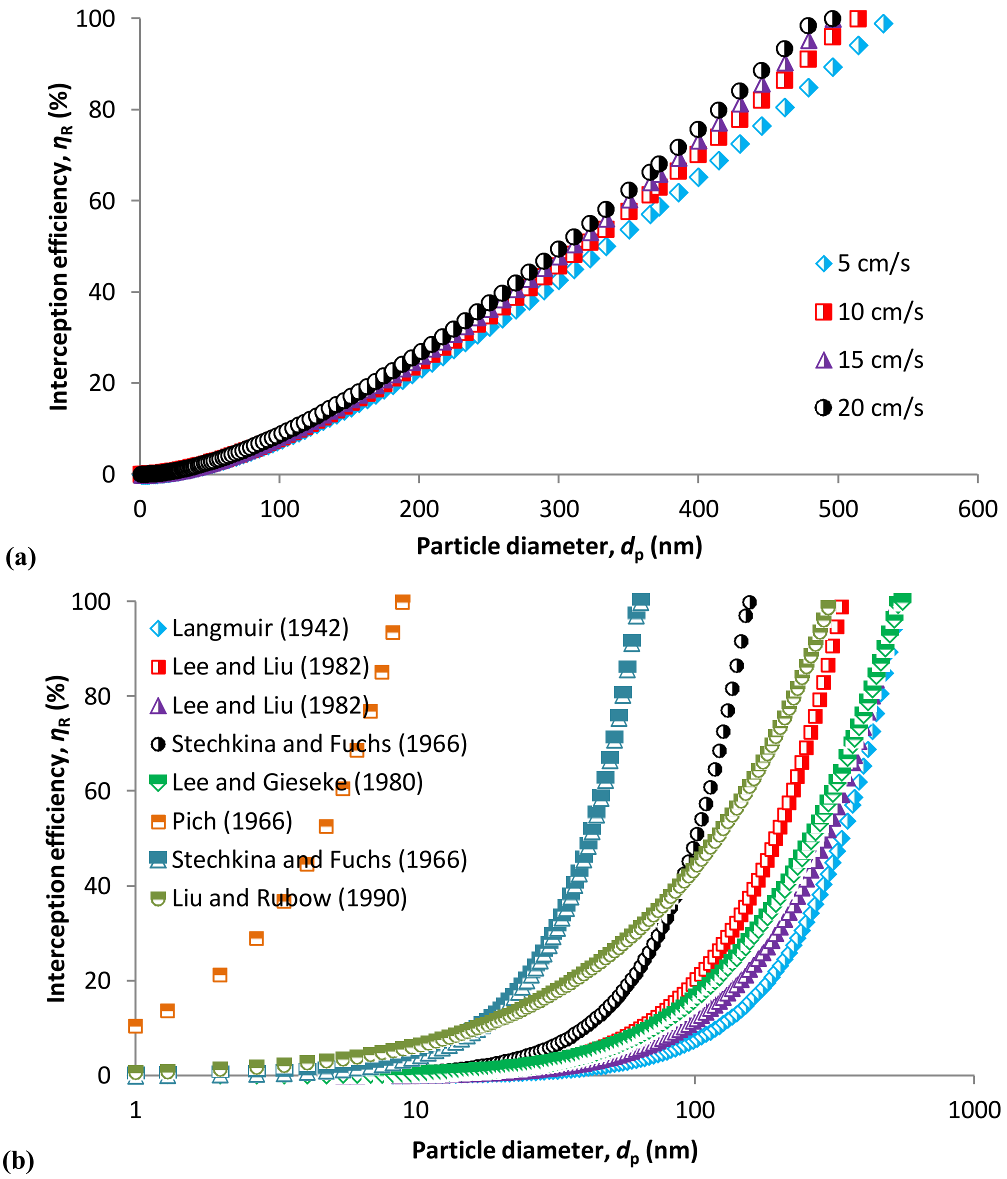


**Figure S3.** Collection efficiency due to interception mechanism based on Langmuir model for different airflow velocities (a) and a comparison of SCE due to interception based on models developed by various researchers (b)

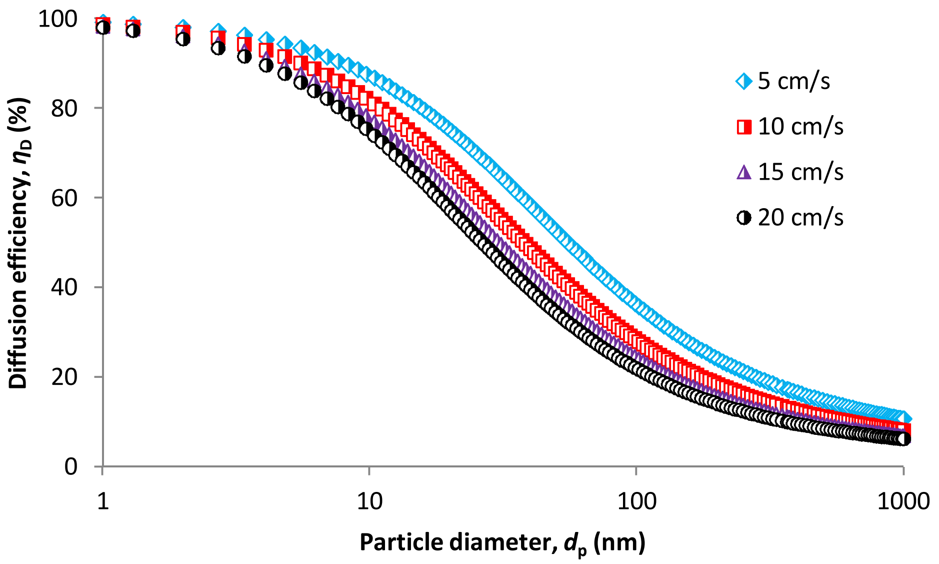

Diffusion efficiency, $\eta_D$ (%)
Particle diameter, $d_p$ (nm)
100
80
60
40
20
0
1
10
100
1000
5 cm/s
10 cm/s
15 cm/s
20 cm/s


(a)

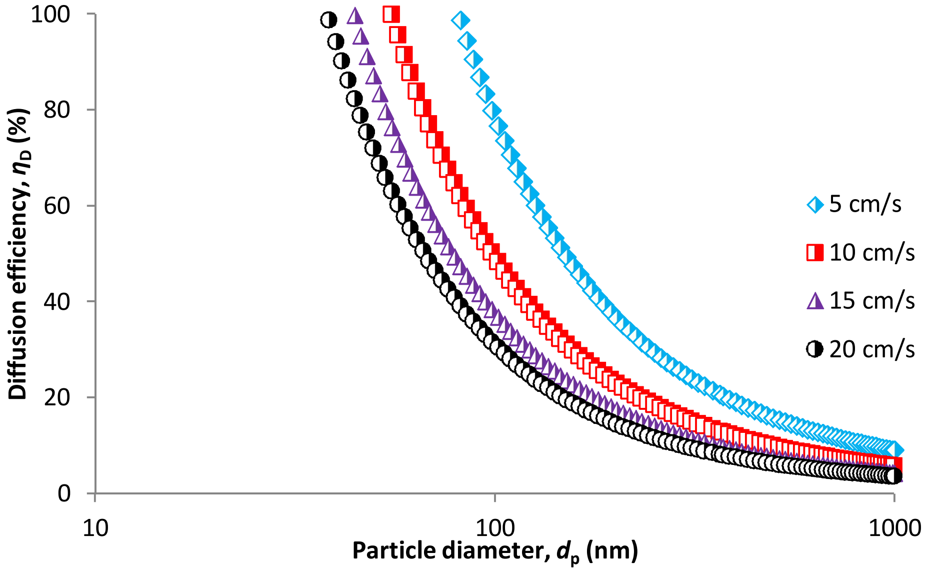

Diffusion efficiency, $\eta_D$ (%)
Particle diameter, $d_p$ (nm)
100
80
60
40
20
0
10
100
1000
5 cm/s
10 cm/s
15 cm/s
20 cm/s


(b)

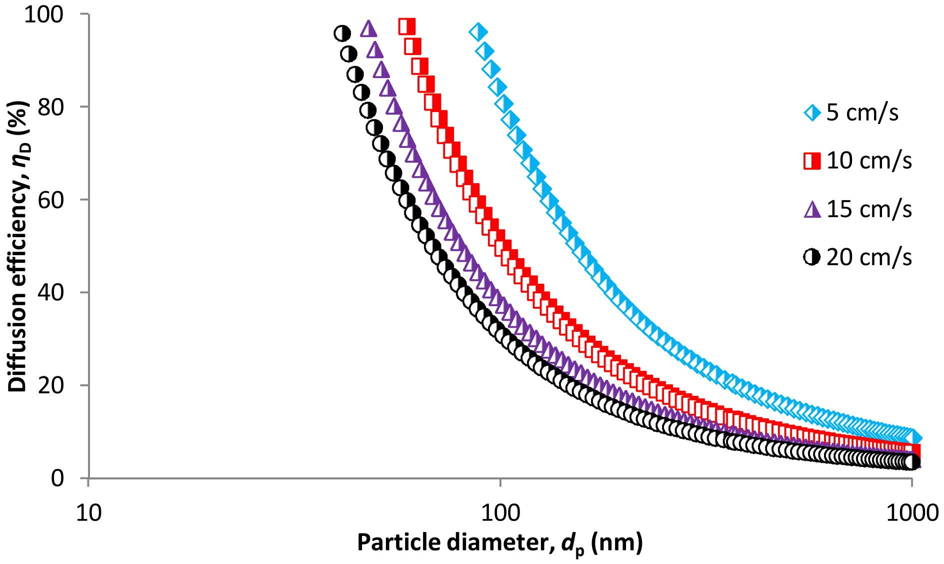

Diffusion efficiency, $\eta_D$ (%)
Particle diameter, $d_p$ (nm)
100
80
60
40
20
0
10
100
1000
5 cm/s
10 cm/s
15 cm/s
20 cm/s


(c)

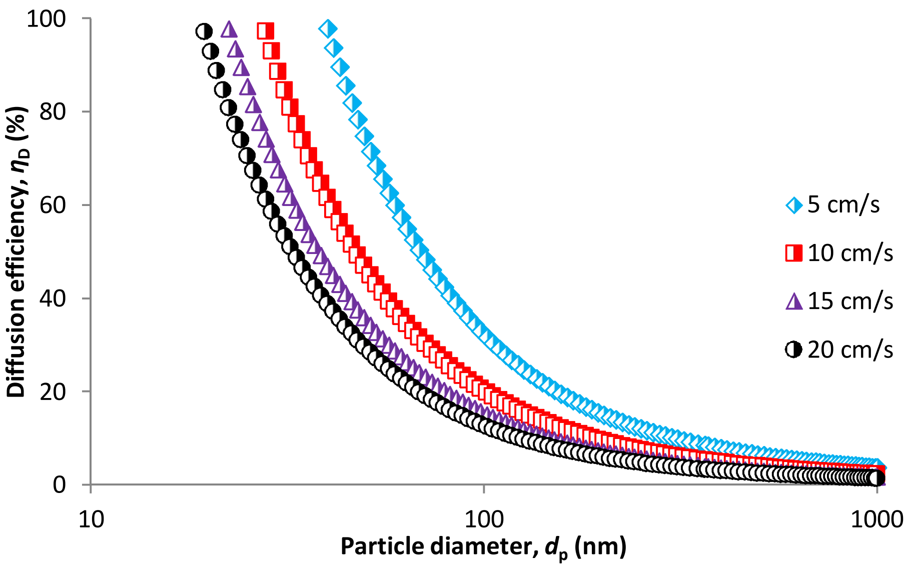


(d)

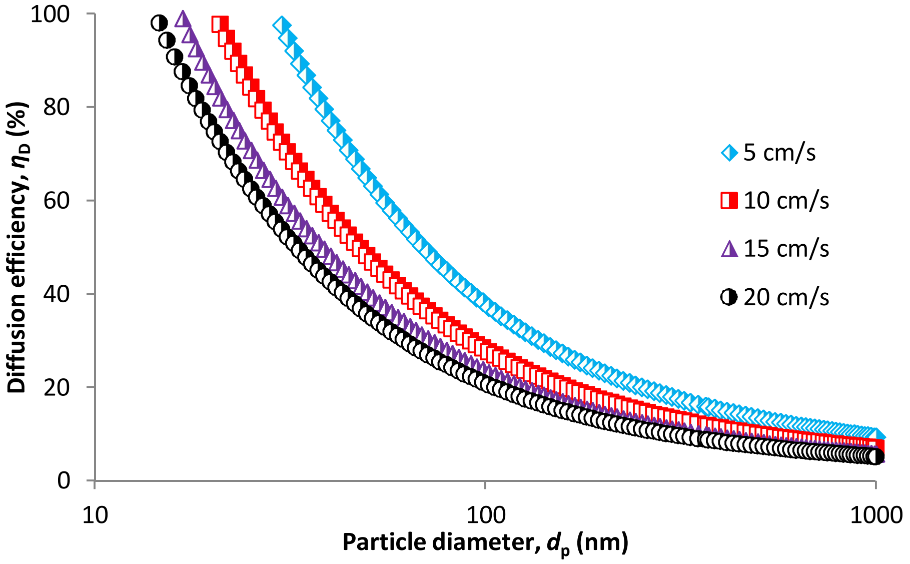


(e)

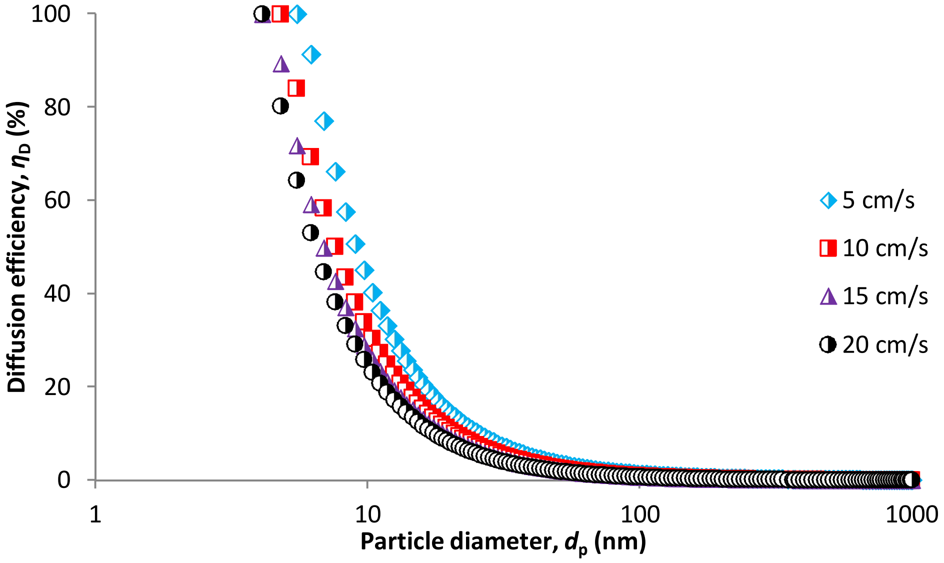


(f)

**Figure S4.** SCE due to diffusion mechanism based on mathematical models developed by Payet et al. (a), Kirsch and Fuchs (b), Stechkina et al. (c), Lee and Liu (d), Wang et al. (e) and Pich (f)

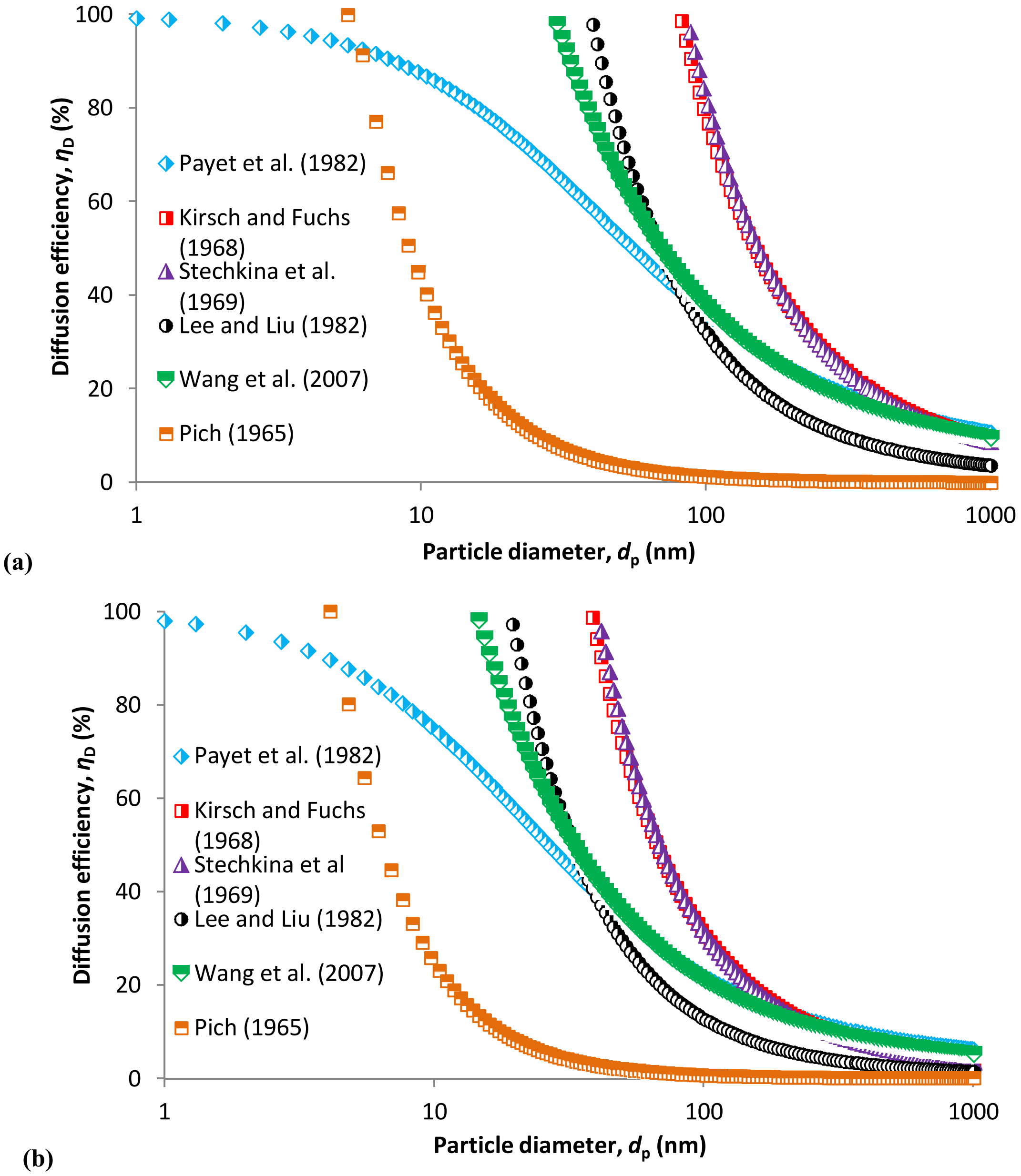


**Figure S5.** Comparison of SCE due to diffusion mechanism based on different models for an airflow velocity of 5 cm/s (a) and 20 cm/s (b)
